# The Starvation Symptom Inventory: Development and Psychometric Properties

**DOI:** 10.3390/nu9090967

**Published:** 2017-09-01

**Authors:** Simona Calugi, Mario Miniati, Chiara Milanese, Massimiliano Sartirana, Marwan El Ghoch, Riccardo Dalle Grave

**Affiliations:** 1 Department of Eating and Weight Disorders, Villa Garda Hospital, Via Montebaldo, 89, 37016 Garda (Verona), Italy; sartirana@adacentroobesitaanoressia.it (M.S.); marwan1979@hotmail.com (M.E.G.); rdalleg@gmail.com (R.D.G.); 2 Department of Clinical and Experimental Medicine, University of Pisa, Via Roma, 67, 56100 Pisa, Italy; mario_miniati@yahoo.it; 3 Department of Neurosciences, Biomedicine and Movement Sciences, University of Verona, 37129 Verona, Italy; chiara.milanese@univr.it

**Keywords:** anorexia nervosa, starvation symptoms, dietary restraint, validity, reliability, factor analysis

## Abstract

**Background**: Starvation symptoms are common in patients with anorexia nervosa, and according to the transdiagnostic cognitive behavioural theory for eating disorders, they contribute to maintaining the eating disorder psychopathology. The aim of this study was therefore to describe the design and validation of the Starvation Symptoms Inventory (SSI); a self-report questionnaire that examines the symptoms of starvation in underweight patients with eating disorders. **Methods**: 150 female patients with anorexia nervosa were recruited, as well as 341 healthy control subjects, 30 not-underweight patients with an eating disorder, and 15 patients with bipolar depressive episodes. The 150 patients completed the Eating Disorder Examination Questionnaire and the Brief Symptom Inventory. All participants rated their starvation symptoms on a continuous Likert-type scale (0–6), and reported the number of days in which they had experienced them in the previous 28 days. **Results**: Principal component analysis identified a single-factor, 15-item scale, which demonstrated good internal consistency (α = 0.91) and test–retest reliability (*r* = 0.90). The SSI global score was significantly correlated with eating disorder and general psychopathology, demonstrating good convergent validity. SSI scores were significantly higher in the anorexia nervosa sample than in the healthy control, not-underweight eating disorder and bipolar depressive episode samples. **Conclusions**: These findings suggest that the SSI is a valid self-report questionnaire that may provide important clinical information regarding symptoms of starvation in patients with anorexia nervosa.

## 1. Introduction

In the classic two-volume *The Biology of Human Starvation*, Keys and colleagues gave a detailed description of the symptoms of dietary restriction and underweight reported by 36 young male volunteers who participated in the Minnesota Starvation Study [1]. The major “starvation symptoms” reported by volunteers included abnormal attitudes and behaviour towards food and eating (e.g., preoccupation with food and eating, ritualistic eating, cookbook and recipe collection), poor emotional and social functioning (e.g., mood liability, social withdrawal, reduction of sexual interest), impaired cognitive performance (e.g., poor concentration), and physiological changes (e.g., heightened satiety, gastrointestinal discomfort, cold intolerance).

The clinical observation that many symptoms reported by these volunteers were similar to those found in patients with anorexia nervosa improved the understanding and management of eating disorders [2]. Indeed, today it is widely accepted that many symptoms that were once attributed to the psychopathology of anorexia nervosa are the mere consequences of calorie restriction and being underweight [3]. 

Moreover, the transdiagnostic cognitive behavioural theory for eating disorders has recently suggested that certain starvation symptoms (e.g., hunger, poor concentration, heightened satiety, dizziness, reduction in rate of weight loss) are interpreted by patients with eating disorders in terms of control over eating, shape, weight or themselves in general [4,5]. It has also been proposed that some individuals with an eating disorder interpret some symptoms of starvation (e.g., dizziness, hunger) as positive signs of being in control—as evidence that they are working hard to achieve their goal of controlling eating, shape, and weight [5]. This hypothesis is supported by two studies showing that patients with eating disorders are significantly more likely to interpret certain starvation symptoms as proof of control than healthy subjects [5,6]. 

Although awareness and assessment of starvation symptoms may be very useful in clinical practice, to our knowledge no instrument is yet available to assess these symptoms in patients with eating disorders. The Self-Starvation Scale, a self-report tool in part adapted by the Yale Food Addiction Scale [7], has been recently validated, but this assesses the compulsive ‘dependence’ on starvation in patients with anorexia nervosa, not the starvation symptoms themselves [8]. 

Hence, we set out to design a novel Starvation Symptom Inventory (SSI) to measure the main symptoms of starvation, and to validate it in anorexia nervosa, not-underweight eating disorder, bipolar depressive episode, and healthy control samples. We hypothesized that patients with anorexia nervosa would have a higher SSI global score compared to not-underweight eating disorder, bipolar depressive episode, and healthy control samples. Moreover, we expected that the healthy control sample would have a significantly lower SSI score than not-underweight eating disorder and bipolar depressive patients and that not-underweight eating disorder patients would have a higher SSI global score than patients with bipolar depressive episodes.

## 2. Methods

### 2.1. Participants

#### 2.1.1. Anorexia Nervosa Sample

The sample comprised 150 female patients meeting the Diagnostic and Statistical Manual of Mental Disorders (DSM-5) diagnostic criteria for anorexia nervosa. DSM-5 diagnosis was conducted by experts in the field using the Italian version of the Eating Disorder Examination (EDE) interview [9]. In total, 128 of the patients were recruited from the inpatient unit of Villa Garda Hospital (northern Italy) and 22 from an outpatient eating disorder clinic serving the Verona area of Italy. 

#### 2.1.2. Not-Underweight Eating Disorder and Bipolar Depressive Episode Samples

A group of 30 not-underweight eating disorder female patients (*n* = 14 with bulimia nervosa; *n* = 4 with binge-eating disorder and overvaluation of shape and weight; and *n* = 12 with other specified feeding or eating disorders), diagnosed by means of the EDE interview, was recruited from outpatient (*n* = 13) and inpatient (*n* = 17) settings. Patients with other specified feeding or eating disorders were excluded from the study if they had atypical anorexia nervosa.

A group of 15 female patients with bipolar disorder was administered the inventory in an outpatient clinic serving the Tuscany area, Italy. A senior psychiatrist made a clinical diagnosis of bipolar with depressive episode using a standard checklist of DSM-5 criteria. Patients with bipolar disorder were excluded if they (i) were underweight (Body Mass Index (BMI) <18.5 kg/m^2^); (ii) met the diagnostic criteria for eating disorder, including binge-eating disorder; and/or (iii) had lost at least 10% of their body weight in the previous 6 months. 

These two groups were included in order to allow analysis of their differences with the anorexia nervosa sample. 

#### 2.1.3. Healthy Control Sample

The healthy control sample comprised 341 female subjects. In total, 217 were recruited from the general population in various community settings and took part in an online survey and 124 were recruited among university students and staff-members, and completed the paper and pencil questionnaire. All healthy control participants self-reported weight and height. Subjects were excluded if they reported a BMI < 18.5 kg/m^2^ (*n* = 20) and/or scored higher or equal to 20 on the validated Italian version of Eating Attitudes Test-26 (EAT-26) [10,11] (*n* = 15) and/or there was a suspicion or diagnosis of eating disorder, as they responded affirmatively to one or both of the following two questions: “Do you have an eating disorder”? and/or “Do you attend a treatment for eating disorders”? (*n* = 1).

The study design was reviewed and approved by the Institutional Review Board of Villa Garda Hospital, Verona. Participants gave a written informed consent for anonymous use of their data for clinical and research purposes. For those under the age of 18 years, additional informed consent was provided by their parents.

### 2.2. Measures

#### 2.2.1. Eating Disorder Examination Questionnaire (EDE-Q)

The EDE-Q [12] is a self-report measure of relevant attitudes and behaviours over the previous 28 days. The items are rated on a 7-point forced-choice format (0–6), with higher scores reflecting greater severity or frequency. Items are grouped into subscales (restraint, eating concern, weight concern, shape concern), and the global score is taken as the mean score of the four subscales. A number of behavioural measures (objective and subjective bingeing, vomiting, laxative use, etc.) was also recorded. A validated Italian version of the EDE-Q 6.0 [13] was used to evaluate the relationship between starvation symptoms and eating disorder psychopathology. 

#### 2.2.2. Brief Symptom Inventory (BSI)

The BSI is a 53-item self-report tool that evaluates psychological distress and psychiatric disorders. The Global Severity Index (GSI) was calculated from scores assigned to participants’ responses on the validated Italian version [14,15]. This scale was used to assess the relationship between starvation symptoms and general psychiatric features.

### 2.3. Tool Design

#### Starvation Symptom Inventory (SSI)

Items for the initial SSI were generated by taking into consideration starvation symptoms recorded in the Minnesota Starvation Study [1], clinical observations from patients’ reports, and multiple discussions among researchers and clinicians specialised in eating disorders. The initial pool of items was discussed among the authors in order to reach a consensus on which to add or remove in order to obtain a comprehensive description of starvation symptoms. This process resulted in 16 items. The structure of the SSI mirrors that of EDE-Q, and participants are asked to provide an estimate of the number of days out of the preceding 28 (four weeks) in which they have experienced these symptoms on a 7-point Likert scale ranging from ‘never’ (0) to ‘always’ (6).

### 2.4. Statistical Analysis

To explore construct validity, principal component analysis (PCA) with oblique (promax) rotation—hypothesizing that any identified factors would be correlated—with Kaiser normalization, was performed separately in anorexia nervosa and healthy control samples [16]. Kaiser–Meier–Olkin (KMO) was used to assess the sampling adequacy. The sampling was considered adequate if KMO was higher than 0.5. To ensure an adequate subject to item ratio, the number of questionnaires to be administered was determined in advance (*n* from 80 and 160), as recommended for principal component analysis [17]. The number of factors to be extracted was defined by inspecting the Scree Plot and considering their interpretability and consistency with the criteria that guided the construction of the tool. Items with factor loadings of 0.40 or greater were considered to load on a given factor. Item–total correlations were also examined for a single factor. 

The following analyses were performed in the anorexia nervosa sample: (i) Cronbach’s alpha was calculated as indicator of internal consistency; and (ii) Pearson product-moment correlations were conducted to assess for convergent validity. The SSI was also administered a second time (one to three weeks after the initial administration) to a subset of the anorexia nervosa sample (*n* = 34), who had yet to begin treatment, to allow for calculation of test–retest reliability.

Additionally, criterion validity was tested using analysis of variance (ANOVA) with Bonferroni post-hoc multiple comparisons, comparing SSI global score in the anorexia nervosa sample with the healthy control, not-underweight eating disorder and bipolar depressive episode samples.

All data were analysed using IBM SPSS Statistics 23.0 (SPSS, Chicago, IL, USA).

## 3. Results

### 3.1. Construct Validity

**Figure 1:**
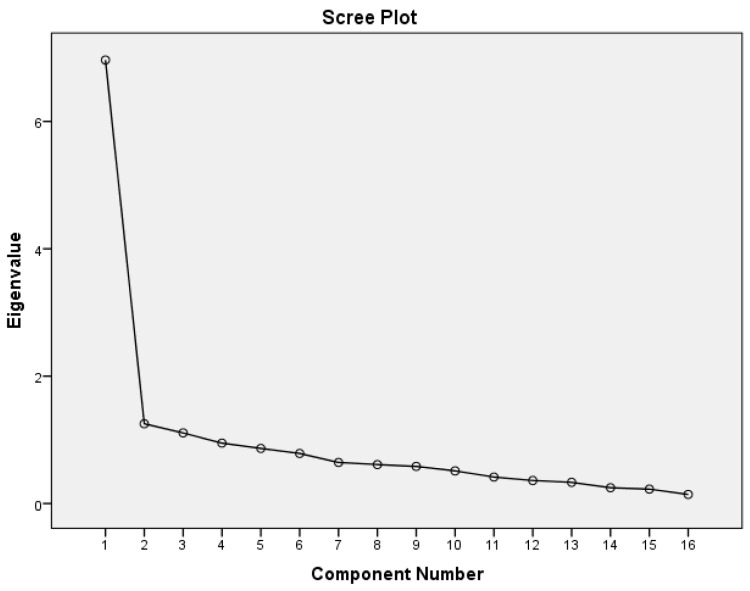
Scree Plot of eigenvalues for the original 16 items of the Starvation Symptom Inventory in the anorexia nervosa sample.

In the anorexia nervosa sample, principal component analysis with oblique rotation revealed three factors with eigenvalues above the 1.00 threshold, accounting for 58.3% of the variance. KMO was 0.88, indicating that principal component analysis was appropriate for 16 items of the SSI. By inspecting the Scree Plot (Figure 1), a clear change in curvature was observed after the 1st factor, suggesting the retention of one factor accounting for 43.5% of the variance. The one-factor solution was also selected as the best in terms of utility and interpretability of the starvation symptoms. 

One item (number 15) of the one-factor solution had factor loadings of less than 0.40 (*r* = 0.16) (see Supplementary Table S1) and was removed from the scale. Table 1 shows the characteristics for the remaining 15 items; the final single-factor solution accounted for 46.4% of the variance, and the factor loadings of the final 15 items ranged from 0.420 to 0.817.

In the healthy control sample, a four-component factor pattern emerged, accounting for 60.9% of the variance. However, the Scree Plot suggested a one-factor solution, accounting for 37.2% of the variance. Item 2 showed a factor loading of less than 0.40 (*r* = 0.021) in the one-factor solution. However, item 15 had a factor loading of 0.572. The remaining factor pattern was the same across anorexia nervosa and healthy control samples.

### 3.2. Reliability

The internal consistency (Cronbach alpha) of the SSI was 0.91. Moreover, the SSI was administered to 34 patients at initial assessment, and again 6–24 days (mean 13.7 days) later, and test–retest reliability of the SSI score was *r* = 0.90, *p* < 0.001.

### 3.3. Correlation Analyses

The mean SSI was not significantly correlated with age (*r* = −0.13, *p* = 0.114) or duration of illness (*r* = −0.060, *p* = 0.467), and only weakly with BMI (*r* = −0.22, *p* = 0.007). In contrast, SSI global score was significantly correlated with EDE-Q global score (*r* = 0.75, *p* < 0.001) and subscales (restraint *r* = 0.60, *p* < 0.001; eating concern *r* = 0.69, *p* < 0.001; weight concern *r* = 0.72, *p* < 0.001; shape concern *r* = 0.74, *p* < 0.001), as well as BSI global score (*r* = 0.82, *p* < 0.001). These results provide evidence for convergent validity. 

### 3.4. Group Mean Comparisons

Table 2 shows baseline characteristics and SSI global score among the four groups. Patients with anorexia nervosa had significantly lower age than patients with bipolar depressive episodes and the healthy control sample, significantly lower body weight and BMI and a higher SSI global score than the other three groups. Moreover, the SSI global score was significantly higher in not-underweight eating disorder patients compared with bipolar depressive episode and healthy control samples. Finally, patients with bipolar depression had a higher SSI global score than healthy controls. The significant difference among the four groups is confirmed when controlling the analysis for age and BMI. 

## 4. Discussions

This study aimed to design and validate the SSI in a group of patients with anorexia nervosa, and to compare their scores with the healthy control, not-underweight eating disorder and bipolar depressive episode samples. 

There were three main findings, the first regarding construct validity. Specifically, PCA in patients with anorexia nervosa indicated that the one-factor solution was the best, accounting for more than 43% of the variance. Similar results were obtained in the healthy control sample. Interestingly, the PCA enabled the identification of one item (namely, “Felt an increase in hunger”) with very low factor loading; these were omitted from the final tool, which therefore comprised 15 items (see Supplementary Table S2). 

The second finding was that the final version of the SSI showed very good internal consistency and test–retest reliability. This indicates that the items measure the same general construct, and that the tool is stable over time. 

Our third finding concerned the convergent and divergent validity. In particular, the SSI global score was significantly associated with eating disorder and general psychopathology, and showed significantly higher scores in the anorexia nervosa sample, as compared with the healthy control, not-underweight eating disorder and bipolar depressive episode samples. This indicates that the starvation symptoms investigated are related to the psychopathology, but are specific for underweight patients. Moreover, the small but significant difference between patients with anorexia nervosa and not-underweight eating disorders could indicate that some symptoms included in the SSI are not specific to starvation, but are instead features of dietary restraint [5].

This study has a number of strengths, including the use of a large sample of treatment-seeking individuals with anorexia nervosa. Moreover, the inclusion of a large healthy control sample and a group of not-underweight patients with eating disorder psychopathology and patients with bipolar depressive episodes enabled examination of the psychometric and clinical validity of the tool. In particular, it allows us to exclude the possibility that some symptoms are related to eating disorder psychopathology or depressive symptomatology rather than starvation. 

However, the study also has limitations. The first concerns the inability to assess the concurrent validity with other existing tools, because no validated instruments to assess symptoms of starvation are available. Furthermore, the relatively limited clinical sample size prevented us from using confirmatory factor analysis or item-response theory analysis to examine the performance of items in greater detail. Moreover, the low number of patients in the not-underweight eating disorder and bipolar depressive episode samples could limit the representativeness of their respective populations. 

## 5. Conclusions 

The SSI appears to be a promising tool for measuring symptoms of starvation in patients with anorexia nervosa; it could be easily integrated into routine clinical practice to assess starvation symptoms in underweight patients with eating disorders, and to assess their changes during the process of weight restoration in those who undergo specialized eating-disorder treatments.

## Figures and Tables

**Table 1:** 15-Item Starvation Symptom Inventory: means (standard deviations), eigenvalues, percentages of variance, factor loadings and item–total correlations in the anorexia nervosa sample.

	Mean (SD) *	Eigenvalue	% Variance	Factor	Item–Total
1. Worried about food?	5.2 (1.7)	6.96	46.4	0.605	0.54
2. Collected recipes, menus or cookbooks?	1.6 (2.1)	1.12	7.5	0.420	0.37
3. Increased your consumption of tea, coffee or spices?	2.7 (2.5)	0.96	6.4	0.555	0.51
4. Felt depressed?	3.8 (2.0)	0.88	5.8	0.817	0.75
5. Felt anxious?	4.4 (1.9)	0.80	5.3	0.799	0.73
6. Felt irritable?	4.1 (1.9)	0.79	5.1	0.755	0.69
7. Had mood swings (between excited and depressed)?	4.2 (1.9)	0.61	4.0	0.763	0.70
8. Stayed away from other people?	3.8 (2.1)	0.60	4.0	0.786	0.72
9. Experienced a loss of concentration?	3.6 (2.1)	0.52	3.4	0.706	0.65
10. Felt apathetic?	3.3 (2.2)	0.42	2.7	0.810	0.75
11. Had disturbed sleep?	3.4 (2.2)	0.39	2.6	0.636	0.58
12. Felt weak?	3.7 (2.1)	0.33	2.2	0.675	0.62
13. Experienced a lack of interest in sex?	4.2 (2.3)	0.26	1.8	0.659	0.59
14. Felt cold?	4.3 (2.0)	0.22	1.5	0.581	0.53
15. Felt full early?	3.8 (2.3)	0.14	0.9	0.499	0.44

* Measured on a Likert-type scale scored from 0–6.

**Table 2:** Baseline characteristics and Starvation Symptoms Inventory (SSI) global score in patients with anorexia nervosa, not-underweight patients with an eating disorder, patients with bipolar depressive episodes and healthy controls.

	Anorexia Nervosa (*n* = 150)	Not-Underweight Eating Disorder (*n* = 30)	Bipolar Depressive Episode (*n* = 15)	Healthy Controls (*n* = 341)	*p*-Value	Post-hoc Comparison
Age (years)	25.2 (9.4)	28.3 (11.6)	32.2 (9.1)	30.0 (9.9)	<0.001	a < c, d
Body weight (kg)	38.8 (5.8)	71.6 (26.8)	64.3 (11.2)	61.2 (9.8)	<0.001	a < b, c, d b > d
Body Mass Index (kg/m^2^)	14.7 (1.8)	26.6 (9.0)	24.1 (2.2)	22.0 (2.7)	<0.001	a < b, c, d b > d
SSI global score	55.1 (20.4)	46.9 (22.9)	26.3 (8.4)	10.4 (9.7)	<0.001	a > b, c, d b > c, d c > d

a = patients with anorexia nervosa; b = patients with not-underweight eating disorder; c = patients with bipolar depressive episodes; d = healthy control sample.

## Supplementary Materials

The following are available online at www.mdpi.com/2072-6643/9/9/967/s1, Table S1: 16-Item Starvation Symptom Inventory: means (standard deviations), eigenvalues, percentages of variance, factor loadings, and item–total correlations in the anorexia nervosa sample, Table S2: Starvation Symptom Inventory (SSI).
