# Supplementary material for: The Starvation Symptom Inventory: Development and Psychometric Properties
Source: Nutrients. 2017 Sep 1;9(9):967. doi: 10.3390/nu9090967 (PMC5622727; doi:10.3390/nu9090967)
Supplement: Supplementary file 1 [file nutrients-09-00967-s001.zip › nutrients-219776-supplementary material.pdf]

Supplementary Table S1. 16-Item Starvation Symptom Inventory: means (standard deviations), eigenvalues, percentages of variance, factor loadings, and item–total correlations in the anorexia nervosa sample.

	Mean (SD)*	Eigenvalue	% variance	Factor	Item–total
1. Worried about food?	5.3 (1.6)	6.96	43.5	0.603	0.54
2. Collected recipes, menus or cookbooks?	1.5 (2.0)	1.25	7.8	0.421	0.37
3. Increased your consumption of tea, coffee or spices?	2.6 (2.6)	1.01	6.9	0.551	0.51
4. Felt depressed?	3.8 (2.0)	0.95	5.9	0.813	0.74
5. Felt anxious?	4.4 (1.9)	0.86	5.4	0.795	0.72
6. Felt irritable?	4.2 (1.9)	0.79	4.9	0.753	0.68
7. Had mood swings (between excited and depressed)?	4.3(1.9)	0.64	4.0	0.763	0.70
8. Stayed away from other people?	3.7 (2.1)	0.61	3.8	0.784	0.72
9. Experienced a loss of concentration?	3.5 (2.2)	0.58	3.6	0.705	0.65
10. Felt apathetic?	3.2 (2.2)	0.51	3.2	0.808	0.75
11. Had disturbed sleep?	3.4 (2.2)	0.42	2.6	0.632	0.57
12. Felt weak?	3.6 (2.1)	0.36	2.2	0.675	0.63
13. Experienced a lack of interest in sex?	4.2 (2.2)	0.33	2.1	0.657	0.59
14. Felt cold?	4.2 (2.0)	0.25	1.5	0.577	0.53
15. Felt an increase in hunger? [&]	1.6 (1.8)	0.23	1.4	0.160	0.13
16. Felt full early?	3.7 (2.3)	0.14	0.9	0.520	0.45

* Measured on a Likert-type scale scored from 0–6. & Removed due to factor loading <0.40

Supplementary Table S2: Starvation Symptom Inventory (SSI)

**STARVATION SYMPTOM INVENTORY**<br>**SSI**	**SURNAME: ………………………………**<br>**NAME: ………………………………………**<br>**DATE: ………………………………………**

INSTRUCTIONS: The following questions are about the past four weeks (28 days). Please read each question carefully and respond to ALL questions. Thank you.

How many times in the last 28 days have you:	Never	1–5 days	6–12 days	13–15 days	16–22 days	23–27 days	Every day
Worried about food?							
Collected recipes, menus or cookbooks?							
Increased your consumption of tea, coffee or spices?							
Felt depressed?							
Felt anxious?							
Felt irritable?							
Had mood swings (between excited and depressed)?							
Stayed away from other people?							
Experienced a loss of concentration?							
Felt apathetic?							
Had disturbed sleep?							
Felt weak?							
Experienced a lack of interest in sex?							
Felt cold?							
Felt full early?							
